# THE EFFECTS OF ROCKER SOLE ON RUNNING KINEMATICS AND WEIGHT-BEARING COMPUTED TOMOGRAPHY: A 3D ANALYSIS STUDY

**DOI:** 10.1590/1413-785220253303e292733

**Published:** 2025-12-01

**Authors:** Rafael Barban Sposeto, Alexandre Leme Godoy-Santos, Albert DaCosta, Leonardo Metsavaht, Gustavo Leporace, Eric Ferkel, Cesar de Cesar Netto

**Affiliations:** 1 Universidade de Sao Paulo (USP), Departamento de Ortopedia e Traumatologia (IOT), Divisao de Pe e Tornozelo, Sao Paulo, SP, Brazil.; 2 Universidade de Sao Paulo (USP), Sao Paulo, SP, Brazil.; 3 Instituto Brasil de Tecnologias da Saude (IBTS), Departamento de Biomecanica Humana, Rio de Janeiro, RJ, Brazil.; 4 Southern California Orthopedic Institute, In Affiliation with UCLA Health, Los Angeles, CA, USA.; 5 Duke University, Durham, North Carolina, USA.

**Keywords:** Kinematics, CT Scan, X Ray, Metatarsalgia, Forefoot, Human, Cinemática, Tomografia Computadorizada, Metatarsalgia, Antepé Humano

## Abstract

Rocker sole footwear can be indicated as a treatment for forefoot load distribution disorders, such as diabetic ulcers, metatarsalgia, and hallux rigidus, among others, with favorable clinical outcomes. Pressure analysis studies on rocker sole footwear highlight results that explain their clinical benefits for forefoot conditions. There is insufficient data in the literature to understand the changes in foot mobility and anatomy caused by rocker sole footwear. This study proposes a methodology using weight-bearing computed tomography (WBCT) to evaluate the anatomical alterations in the forefoot associated with rocker sole footwear. The goal is to investigate morphological changes in the forefoot that explain the clinically established kinetic and kinematic effects of such footwear. Biomechanical analysis potentially enhances the understanding of kinetic and kinematic findings without the bias of rocker sole position or magnitude changes. *Level of Evidence V; Expert Opinion.*

## RESUMO

Calçados com solado rocker podem ser indicados como tratamento para distúrbios de distribuição de carga no antepé, como úlceras diabéticas, metatarsalgia e hálux rígido, entre outros, com resultados favoráveis na evolução clínica. Estudos da avaliação de pressão sob o pé com o uso de calçado com solado rocker evidenciam resultados, que explicam a vantagem clínica do uso desses calçados para o antepé. Não há dados suficientes na literatura para compreendermos quais são as alterações na mobilidade e anatomia dos pés impostas pelos calçados com solados rocker. Propomos nesse artigo uma metodologia de mensuração com tomografia com carga (TCC), avaliar as alterações anatômicas do antepé associadas ao uso do calçado com solado rocker. O objetivo é investigar as alterações morfológicas no antepé que justifiquem os efeitos clínicos, cinéticos e cinemáticos, já estabelecidos na literatura. A análise biocinética potencialmente facilita a compreensão cinética e cinemática dos achados anatômicos, sem o viés da mudança da posição e da magnitude do rocker no solado. *Nível de Evidência V; Opinião do Especialista.*

## INTRODUCTION

Rocker sole footwear, referred to in the literature as "rocker bottom" or "rocker profile,"^1,2^ demonstrates mechanical advantages in redistributing forefoot load, reducing localized overload. As a result, this footwear is frequently used to treat diabetic foot, hallux rigidus, metatarsalgia, sesamoiditis, stress fractures in athletes, and other conditions.^3-5^

These shoes are widely used for treating foot and ankle pathologies, recreational activities, and sports, particularly running. Rocker soles introduce modifications, such as an increment in the plantar volume proximal to the metatarsal heads, which creates a pivot point during the stance phase, altering mechanical and biokinetics properties.

Despite their shared characteristic of sole rigidity, rocker footwear varies significantly in other features. The plantar volume may be angular or rounded, differing in magnitude and anterior or posterior placement along the sole.^4^ These variations affect their mechanical properties and the degree of load redistribution, reducing forefoot pressure.^6^

There are three rocker sole positions: forefoot, hindfoot (negative heel), and double. Forefoot rockers are recommended to decrease overload in this region, hindfoot rockers accommodate tibiotalar joint stiffness, and double rockers reduce midfoot overload.^4^

In forefoot rockers, the position and the magnitude can be quantified. The magnitude is described in degrees (for angular soles) or curvature radius (for rounded soles).^1,7^ The position is defined as a percentage, calculated by dividing the distance from the rocker apex to the sole's anterior edge by the total sole length.

Changes in rocker position and shape yield varied pressure redistribution outcomes. For forefoot conditions, most rockers are positioned between 50% and 60%, with angles ranging from 20° to 30°.^1,4,7^

## INDICATIONS

Rocker sole footwear is frequently employed as an offloading strategy in the treatment of diabetic foot, yielding favorable clinical outcomes in ulcer management.^3-5^

Due to its mechanical properties, likely related to restricting forefoot and tibiotalar joint movement in the sagittal plane, rocker footwear is also indicated for conservative management of forefoot conditions such as hallux rigidus, sesamoiditis, metatarsalgia, Morton's neuroma, stress fractures, metatarsophalangeal osteoarthritis, and rheumatoid forefoot deformities.^8,9^

For hindfoot and ankle conditions, rocker soles accommodate movement limitations caused by tibiotalar osteoarthritis and improve gait patterns in patients with tibiotalar arthrodesis.^7^

## ROCKER SOLE MECHANICS

The theoretical explanation for rocker sole functionality is based on the pivot created by the sole's plantar volume. This pivot forces gait progression as body weight transfers over it, decreasing the range of dorsiflexion of the metatarsophalangeal joints and reducing pressure on the metatarsal heads. Thus, the rocker effect primarily occurs during the terminal stance phase.^10,11^

Numerous studies confirm reduced forefoot pressure during gait with rocker footwear. Fuller et al.^3^ reported a 21% reduction in peak pressure in asymptomatic individuals, while Brown et al.^4^ observed reductions in pressure under the first, second, and third metatarsal heads of 6.97%, 54.44%, and 25.87%, respectively, compared to conventional footwear.

The kinematics and kinetics of gait produce mixed results, showing few changes when comparing rocker soles with conventional shoes.^2,11,12^ Myers et al.^11^ observed a two-step per minute increase with rockers but found no changes in step length, gait speed, or stance duration.

Boyer and Andriacchi^2^ analyzed the running patterns of 19 healthy volunteers using conventional and rocker forefoot shoes. They found greater ankle dorsiflexion during initial contact and mid-stance, with lower running speeds in the rocker group but no significant kinetic or kinematic changes in the knees or hips.

Van Bogard et al.^12^ studied the gait of 40 healthy individuals wearing conventional and rocker forefoot shoes. They noted statistically significant differences, including a more neutral pelvic position in the sagittal plane, increased hip extension during mid and terminal stance phases, greater knee flexion at initial contact, higher ankle dorsiflexion during initial stance, and increased plantarflexion during push-off. No changes in walking speed were observed, though cadence increased, and step length decreased in the rocker group.

Differences in rocker position and magnitude across studies may explain the variability in findings.^13^

Despite descriptions in the literature of the rocker sole's impact on reducing forefoot pressure and gait kinematics, there is no documentation of sagittal plane movement changes in the metatarsophalangeal joints or metatarsal head positioning. This information is important to anatomically justify changes in forefoot pressure and to be able to plan the positioning and magnitude of the rocker individually in specific cases.

Assessing sagittal plane joint positions under load is challenging with radiography due to metatarsal and phalangeal overlap.^14-17^ Radiographic evaluations lack precision due to their bidimensional nature, leading to interpretative errors.^15,18^

Weight-bearing computed tomography (WBCT) provides the advantage of sectional imaging with submillimeter cuts, improving precision and enabling three-dimensional visualization of the metatarsophalangeal joints without overlap, while maintaining physiological load.^16-21^

The authors of this article proposed an ongoing prospective comparative study to evaluate differences in forefoot positioning among volunteers without foot deformities or complaints, using rocker sole footwear during simulated push-off. The objective is to identify and quantify changes in the positioning of the metatarsophalangeal joints during the use of rocker sole footwear, assessed through weight-bearing computed tomography (WBCT) in conjunction with biomechanical analysis. The study was approved by the ethics committee, number 7.021.305. The Informed Consent Form will be explained and applied to the participants.

## TOMOGRAPHIC ASSESSMENT

We utilized the Cone Beam CT LineUP scanner, a cone-beam CT from CurveBeam® (Hatfield, PA, USA), with a field of view of 20 cm height and 20–35 cm diameter. The radiation dose per scan was 5 μSv.

The acquisition follows the standard protocol of the device for foot imaging. The protocol includes settings of 120 kVp, 5.0 mA, 43.2 mAs, a rotation time of 26 seconds, a CTDI vol of 2.717 mGy, and a dose-area product of 15.01 dGy*cm² with a 12-millisecond pulse. We set the increment at 0 mm, the collimation detector fixed at 5% as per factory calibration, a slice thickness of 0.31 mm ± 0.5 mm with a 0.3 mm interval between slices, and a field of view adjusted to 35 cm in diameter and 20.9 cm in height. No filters were applied during acquisition.

For the analysis of the tomography scans, we used the CubeVue© software version 3.7.0.3, developed by CurveBeam® (Hatfield, PA, USA). This program imports the DICOM images generated by the WBCT and provides a comprehensive analysis of the foot.

We positioned the volunteers to simulate the push-off phase of gait, ensuring that the evaluated foot was placed in 15° of ankle plantarflexion under load, while the contralateral foot remained in a plantigrade position, symmetrically distributing the load.

**Figure 1:**
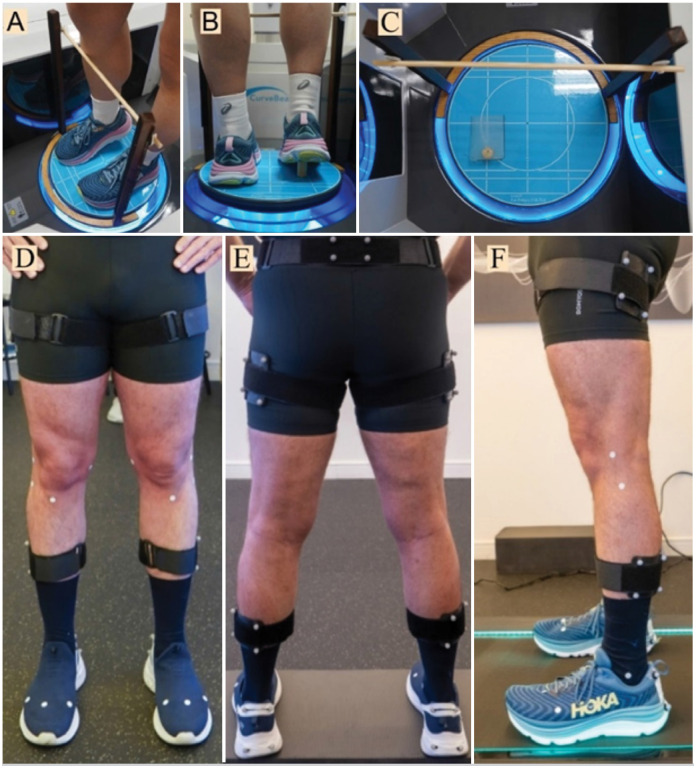
Positioning in WBCT and markers for biomechanical evaluation of the lower limbs.

Source: Images from the author's personal archive. A. Anterior portion of the leg in contact with the anterior positioner, facilitating the reference for the ankle plantar flexion. B. Maintenance of the simulated push-off position with the assistance of the posterior wedge. C. View of the anterior positioning support and the 15° elevated posterior wedge. D. Placement of markers for the biomechanical evaluation on the anterior portion of the lower limbs. E. Placement of markers for the biomechanical evaluation on the posterior portion of the lower limbs. F. Placement of markers for the biomechanical evaluation of the lower limbs in a lateral view.

To maintain this position throughout the scan, we prepared an anterior support in contact with the leg simulating the push-off, combined with a 15° wedge placed under the hindfoot. The anterior support was adjustable vertically to accommodate the volunteer's height, ensuring consistent contact and position maintenance (Figure 1).

The feet were positioned parallel to each other, with the second ray aligned to the gait axis.

Each foot was evaluated under three conditions:

• Barefoot (Barefoot Group - BG): The volunteer was assessed barefoot bilaterally.
• Conventional Sole Footwear (Conventional Group - CG): The volunteer wore bilateral flat, flexible sole shoes.
• Rocker Sole Footwear (Rocker Group - RG): The volunteer wore bilateral rocker sole shoes.

Following this series of scans, the contralateral foot underwent the same protocol, totaling six scans per volunteer. The total radiation exposure of 0.5 mrem per individual was deemed safe, as reported by Kim et al.^22^

The tomographic scan of the foot in a plantigrade position was obtained during the contralateral foot's push-off simulation, allowing for comparisons between plantigrade and push-off positions.

For the CG (Conventional Group), we used a shoe with a flat and flexible sole (Ever Way®, Marvin model). For the RG (Rocker Group), we used a shoe with a rounded rocker sole, an apex located at 60% of the sole's length, a curvature radius of 15.5 cm, a sole height of 30 mm, a 6 mm drop, and a rigid construction (HOKA®, Gaviota 5 model).

We assessed the second and third metatarsophalangeal joints (M2 and M3), as these form the intermediate column of the foot. This segment is more stable proximally in the tarsometatarsal joints, making it less susceptible to minor pronation and supination variations that could affect the positioning of the metatarsophalangeal joints during push-off.^23-25^ This stability ensures more reproducible measurements.

**Figure 2:**
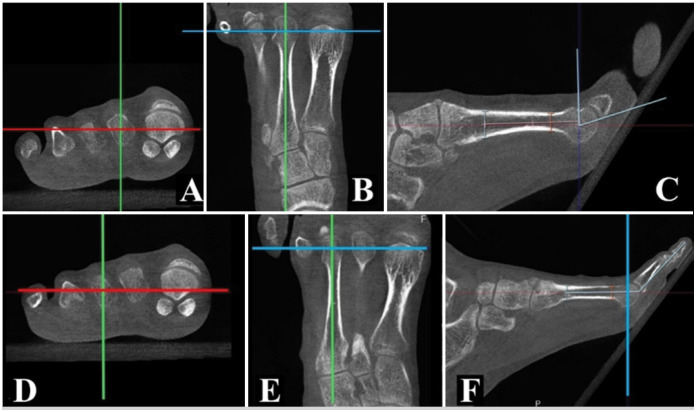
Acquisition of tomographic measurements. Green line – representation of the sagittal plane. Red line – representation of the axial plane. Blue line – representation of the coronal plane.

Source: Images from the author's personal archive. A. Coronal plane, center of the 2nd metatarsal head located. The sagittal plane is perpendicular to the ground. B. Axial plane, center of the 2nd metatarsal head located. The sagittal plane is aligned with the metatarsal axis. C. Sagittal plane, measurement of the angle from the center of the metatarsal head with a line perpendicular to the axis and another line at the most plantar point of the proximal phalanx joint. D. Coronal plane, center of the 3rd metatarsal head located. The sagittal plane is perpendicular to the ground. E. Axial plane, center of the 3rd metatarsal head located. The sagittal plane is aligned with the metatarsal axis. F. Sagittal plane, measurement of the angle between the metatarsal axis and the line connecting the most distal point of the distal phalanx to the point where the metatarsal axis crosses the head.

With the acquired images, we will perform the following measurements:

• Metatarsal Articular Coverage Angle (MACA): Measured in the sagittal plane perpendicular to the ground, passing through the center point of the metatarsal head. The angle is formed by a line perpendicular to the metatarsal's longitudinal axis at the head center and a line connecting this point to the most plantar articular point of the proximal phalanx (Figure 2A, B, C).
• Metatarsophalangeal Joint Extension Angle (MJEA): Measured in the sagittal plane perpendicular to the ground, passing through the metatarsal head center. The angle is formed by the metatarsal's longitudinal axis and a line connecting the most distal point of the distal phalanx to the intersection of the metatarsal axis at the head center (Figure 2D, E, F).

These tomographic measurements aim to assess the mobility of the metatarsophalangeal joint in isolation (MACA) and the angular result of the combined mobility of the metatarsophalangeal and interphalangeal joints (MJEA).

## BIOMECHANICAL ASSESSMENT

Kinematic data are collected using a motion analysis system consisting of eight high-speed cameras (Vero 1.3, Vicon, Oxford, UK), sampling at 100 Hz for the walking test and 250 Hz for the running test. The data are filtered with a Butterworth low-pass filter, with a cutoff frequency defined by the residuals analysis of Winter. 3D angles are calculated according to Grood and Suntay.^26^ All biomechanical data are processed using custom routines in Matlab 2015 software (MathWorks, Natick, USA).

**Figure 3:**
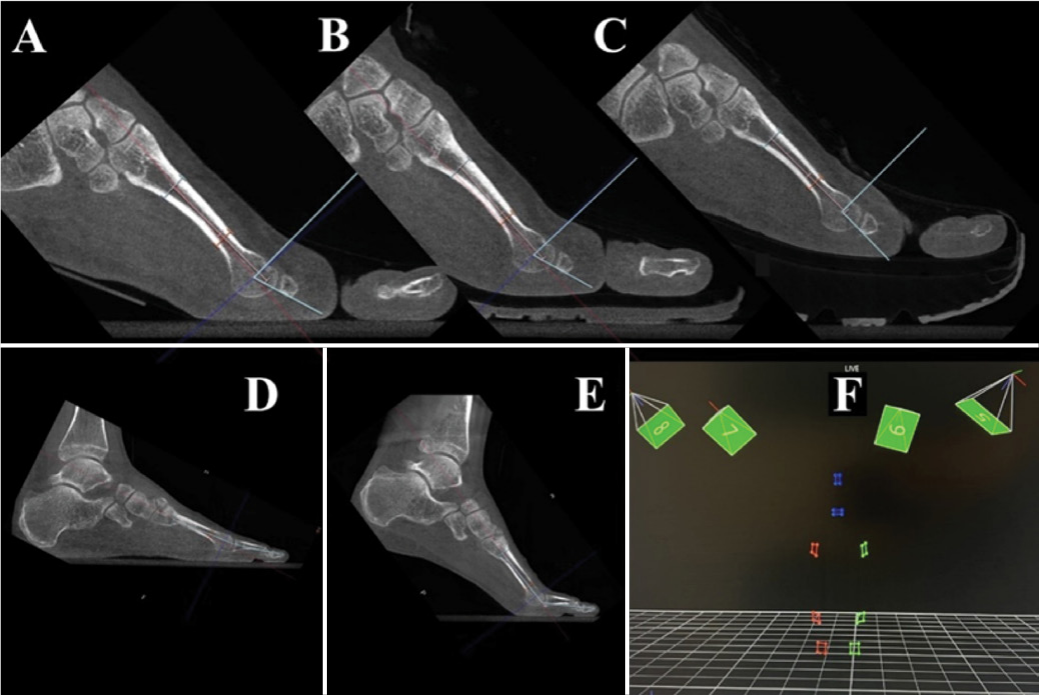
Examples of images obtained in weight-bearing tomographic and biomechanical evaluations.

Source: Images from the author's personal archive. A. Metatarsal articular coverage angle: simulated push-off barefoot, measured at 68°. B. Metatarsal articular coverage angle: simulated push-off with conventional footwear, measured at 72.3°. C. Metatarsal articular coverage angle: simulated push-off with rocker footwear, measured at 90.6°. D. Metatarsophalangeal joint extension angle: barefoot plantigrade. E. Metatarsophalangeal joint extension angle: simulated push-off barefoot. F. Three-dimensional representation of trunk positioning in the lower limbs during biomechanical evaluation.

A marker setup consisting of rigid plates, each with four markers, is positioned on the participants’ thighs and legs (bilaterally). Rigid plates with four markers each are placed on the posterior region of the pelvis (between the posterior superior iliac spines) and on the posterior trunk (at the height of the 10th thoracic vertebra). Four reflective markers are also placed on the posterior part of the participants’ shoes, bilaterally (Figures 1 and 3).

After placing the markers, a static capture is performed for each participant. For this, adhesive markers are applied bilaterally at the following anatomical reference points: the heads of the first and fifth metatarsals, between the heads of the second and third metatarsals, lateral malleolus, medial malleolus, fibular head, tibial tuberosity, medial femoral condyle, lateral femoral condyle, anterior superior iliac spines, xiphoid process, between the clavicles, and at the spinous process of the 7th cervical vertebra.

A pointer with two markers is used to identify these anatomical reference points, following the Calibrated Anatomical Systems Technique (CAST),^27^ thus establishing the participants’ orthostatic posture for calibration and obtaining anthropometric and inertial parameters. After static capture, a functional calibration capture is performed to calculate the hip and knee joint centers.^28,29^

After the calibrations, the running test is conducted with the first shoe, followed by the second shoe.

The Foot Velocity Algorithm^30^ is used to determine the stance and swing phases of each cycle. The Altman and Davis method^31^ is used to check the initial foot contact pattern with the ground.

The running test is performed on a treadmill (Master Top 18, Inbramed, Porto Alegre, Brazil). The participant selects their preferred running speed for the test.

Before the run, a warm-up of approximately 3 minutes of walking at 5.5 km/h is performed. Then, the speed is increased to the running pace, where the participant warms up for about 3 minutes, and a 1-minute running trial is then collected.

During the 3D kinematic assessment, male participants wear shorts or briefs, and female participants wear shorts and a sports top. Each participant performs the walking and running tests with two types of shoes: rocker shoes and conventional shoes, in this order.

With this weight-bearing tomography protocol and biomechanical evaluation, we aim to investigate the functional mechanism of the clinical improvement observed with the use of rocker shoes. The literature suggests that there is less mobility in the metatarsophalangeal joints, as the volume of the rocker proximal to them induces early push-off. Our hypothesis is that WBCT will show that there is less mobility in the metatarsophalangeal joints with the rocker shoe compared to barefoot walking and conventional shoes.

## SUMMARY

The use of rocker sole footwear as a treatment for forefoot load distribution disorders, such as diabetic ulcers, metatarsalgia, and hallux rigidus, among others,^3-5^ has shown favorable results in clinical progression.^32^ Studies evaluating pressure under the foot with rocker sole footwear highlight results that explain the clinical advantage of using these shoes for the forefoot.^2-4,11^

There is insufficient data in the literature to fully understand the changes in foot mobility and anatomy imposed by rocker sole footwear. In this article, we propose a methodology using weight-bearing computed tomography (WBCT) to assess the anatomical changes in the forefoot associated with the use of rocker sole footwear, aiming to find a morphological explanation for the clinical, kinetic, and kinematic effects already established in the literature.

WBCT is an accurate imaging method^17,21^ that would allow us to assess the joint relationship of the five metatarsophalangeal joints, as well as the position of the metatarsals during the push-off phase in individuals wearing rocker sole footwear.

Biomechanical evaluation, performed alongside WBCT using the same sample and footwear, potentially facilitates the understanding of the kinetic and kinematic aspects of the anatomical findings, without the bias of changes in the position and magnitude of the rocker in the sole.
